# Blood Sugar Lowering Effect of *Coccinia grandis* (L.) J. Voigt: Path for a New Drug for Diabetes Mellitus

**DOI:** 10.1155/2011/978762

**Published:** 2011-07-21

**Authors:** M. A. A. K. Munasinghe, C. Abeysena, I. S. Yaddehige, T. Vidanapathirana, K. P. B. Piyumal

**Affiliations:** ^1^General Hospital, Matara, Sri Lanka; ^2^Department of Public Health, Faculty of Medicine, University of Kelaniya, Ragama, Sri Lanka; ^3^Lady Ridgeway Hospital, Colombo, Sri Lanka; ^4^Peripheral Hospital, Kirinda, Thissamaharama, Sri Lanka

## Abstract

*Background*. Role of herbs in the management and control of diabetes has emerged fast over the years. We assessed the efficacy of *Coccinia grandis* (locally known as Ken, Kovakka) leaves as a hypoglycemic agent. *Methods*. Double-blind phase I clinical trial was conducted at the general hospital and a private hospital in Matara in August 2009. All the participants were given a common meal for dinner, and they maintained a 10-hour fasting period. Sixty-one healthy volunteers were given a meal containing 20 g of leaves of *Coccinia grandis* which was mixed with a measured amount of scraped coconut and table salt for breakfast, and other 61 were given the placebo meal which also contained scraped coconut and salt. Glucose tolerance test was performed blindly for the two groups. Mixed factorial design analysis of variance and student's *t*-test were applied. *Results*. Overall blood sugar levels of the experimental group were also significantly lower than those of the control group (F(1,117) 5.56, *P* < 0.05). Increase in the blood sugar levels from fasting to one hour (F(1,117) 6.77, *P* < 0.05) and two hours (F(1,117) 5.28, *P* < 0.05) postprandially was statistically significant for participants who were in the control group than those of in the experimental group. The mean difference of postprandial blood sugar levels (mg/dL) after one hour (20.2, 95% confidence interval, 4.81 to 35.5) and two hours (11.46, 95% confidence interval; 1.03 to 21.9) was statistically significant between the two groups. *Conclusions*. *Coccinia grandis* has a blood sugar lowering effect. However further studies are needed to validate our findings.

## 1. Introduction

It is a well-known fact that throughout the world about 180 million people suffer from diabetes and its dreadful complications [1]. Therefore, new concepts in the management of diabetes have aroused a curiosity among doctors as well as among the patients throughout the world. Role of herbs in the management and control of diabetes has emerged fast over the years with the discovery of hypoglycemic effect of Bitter Melon (*Mormodica charantia*) [2, 3]. Currently the extracts of these plants are used as adjuvant therapy with other medications in many eastern countries for the treatment of diabetes. 

Ken (*Coccinia grandis*) has been used in ayurvedic medicine in Sri Lanka and India to treat diabetes from ancient times [4]. *Coccinia grandis *is also known by the synonyms* Coccinia indica* and *Coccinia cordifolia *[5]. Young leaves and long slender stem tops of the plant are cooked and eaten as a potherb or added to soups. Young and tender green fruits are used either as raw salads or cooked and added to curries. Our attention was focused on this herb, and further investigations revealed that, despite the absence of any scientific evidence, Sri Lankans are well convinced about its hypoglycemic effects. As no publications were found in the literature review regarding the hypoglycemic effect of this herb on healthy volunteers, this study was designed to assess the efficacy of leaves of* Coccinia grandis* as a hypoglycaemic agent in healthy individuals and to identify common short-term adverse effects. However there were two publications regarding hypoglycemic effect of this herb on diabetic patients [6, 7].

## 2. Materials and Methods

This double-blind phase I clinical trial was conducted at the general hospital and a private hospital in Matara using 122 healthy volunteers in August 2009. The volunteers were of 18–55 age range who were nonsmokers and were not on regular medication or alcohol. Pregnant women were excluded from the study. Procedure was explained to each individual, and informed written consent was obtained to participate in the trial. Demographic information was taken on the previous day, and all of them were given a common meal for dinner and 10 hours fasting was maintained. Randomization was performed by tossing a coin in each centre to select the two groups.

Each test meal contained 20 g of leaves of *Coccinia grandis* which were mixed with a measured amount of scraped coconut and table salt. Each sample was prepared in a similar manner. Twenty grams of leaves of *Erythrina indica* (locally known as Erabadu) were mixed with a measured amount of scraped coconut and table salt and given as placebo.* Erythrina indica *is a kind of edible leaves commonly used by Sri Lankans as a salad or tempered leaves. We decided to use *Erythrina indica* because of its similarity in color and taste to of *Coccinia grandis,* and it was not known to cause any adverse effects such as hypoglycemia. All participants were asked to consume the green leaves completely under observation. Sixty-one participants were given a meal containing the testing substance for breakfast, and other 61 were given the placebo meal. The participants were unaware of the type of the meal they received. All of them were given 75 grams of glucose in 300 mL of water and asked to drink it within 20 minutes under observation. Venous blood was drawn after one hour and two hours (2 mL in each occasion) as in oral glucose tolerance test (OGTT) by three trained nurses. Blood samples were analyzed at the laboratory of a private hospital. Medical Laboratory Technologist (MLT) who analyzed the blood sugar level was unaware of the group of allocation of the two treatment regimes. A self-administered questionnaire was used for assessing side effects. 

Data was analyzed using SPSS.16. Student's *t*-test was applied for the blood sugar levels between the two groups before and after the intervention. As this study was involving data collection over the time, a mixed factorial design analysis of variance was applied. Two-tailed *P* value of less than  0.05 was considered as statistically significant. The Ethics Review Committee of the Faculty of Medicine, University of Kelaniya, Sri Lanka granted the ethical approval. 

## 3. Results

While sixty-one participants of the total (50%) received *Coccinia grandis* (the testing substance), the rest of the participants (61) received control (*Erythrina indica*). Among the experimental group 31 (50.8%) were males but only 17 (28%) were males in the control group. The mean age of experimental group was 33 (SD ± 11) years, and the corresponding figure was 37 (SD ± 12) years for the control group. Postprandial blood sugar levels after one hour were found to be missing in one participant from each group, and also sugar levels after two hours were found to be missing in one participant from the control group as well as from two participants of the experimental group.

**Figure 1:**
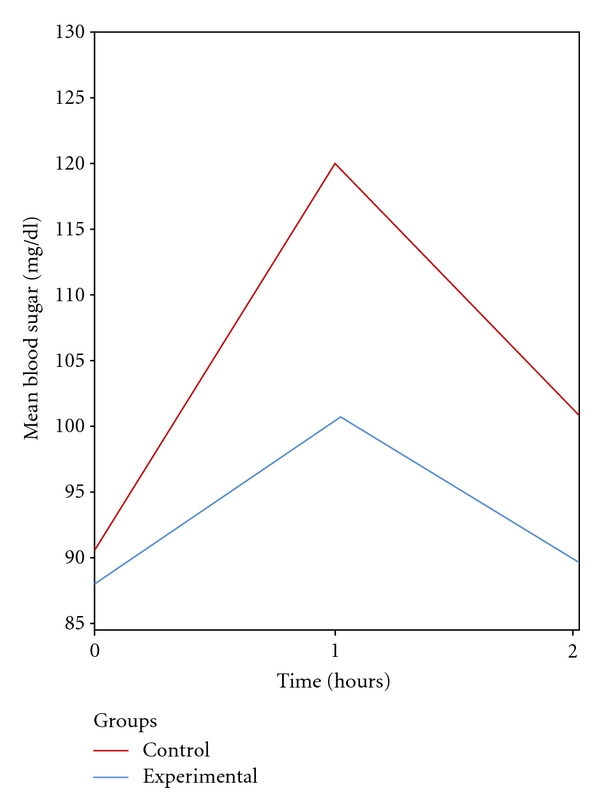
Mean blood sugar levels for experimental and control groups before and after intervention.

As shown in the Figure 1, postprandial blood sugar levels after one hour and two hours were higher in both experimental and control groups. In contrast, the participants in the experimental group showed lower postprandial blood sugar levels after one hour and two hours, respectively. A mixed design analysis of variance showed that this interaction between intervention and postprandial blood sugar levels was significant (F(1.48,173.7) 5.25, *P* < 0.001, partial eta squared = 0.043). An interaction contrast showed that the increase in blood sugar levels from fasting to one hour postprandial was statistically significant for participants who were in the control group than for those in the experimental group (F(1,117) 6.77, *P* < 0.05, partial eta squared = 0.055). A further contrast revealed that the increase in blood sugar levels from fasting to two hours postprandially was statistically significant for participants who were in the control group than in the experimental group (F(1,117) 5.28, *P* < 0.05, partial eta squared = 0.043). Overall blood sugar levels in the experimental group were also significantly lower than in the control group (F(1,117) 5.56, *P* < 0.05, partial eta squared = 0.045). As shown in Table 1, the difference between the fasting blood sugar levels in the two groups were not statistically significant. In contrast, the postprandial blood sugar levels after one hour and two hours were statistically significant between the experimental and control groups.

Nausea, headache, and drowsiness were experienced by 1 (1.7%), 3 (5%), and 17 (29%) participants in the experimental group, respectively, and 2 (3.3%), 3 (5%), and 25 (41.6%) in the control group. Sweating, tremor, pounding heartbeat, pallor, or cold extremities were not reported from any participant.

## 4. Discussion

Our study showed that overall blood sugar levels in the experimental group were significantly lower than those in the control group. The study also showed that the increase in blood sugar levels from fasting to one hour postprandially was statistically significant for participants who were in the control group than for those in the experimental group. The same statistical significance was observed in the two-hour postprandial blood sugar levels. Further, the difference between the blood sugar levels at one hour postprandially (19.5 mg/dL) was more than that at two hours postprandially (11.5 mg/dL). Our findings indicated that raw Ken (*Coccinia grandis*) leaves lowered the postprandial blood sugar levels proving the herb improving the glucose tolerance. Mechanism of action by which the glucose tolerance was improved is to be studied by further research. This can happen at various steps which results in the increased glucose tolerance. Reduction of sugar absorption from the gut, increased insulin production from the pancreas, reduction of release of glucose from the liver, increasing glucose uptake by fat and muscle cells are probable mechanisms which may be involved. Some studies have suggested that suppression of glucose-6-phosphatase [8] increases the activity of glycogen syntheses [9], and it is partly responsible for this. 

We decided to use *Erythrina indica* as a placebo because it has no hypoglycemic effect on humans [10, 11]. Even if it has any hypoglycemic effects, it does not interfere with our results, because we have shown a strong statistical significance in the experimental group using *Coccinia grandis* on its hypoglycemic effects.

We selected 122 healthy volunteers dividing them into two groups which were comparable in terms of age and sex minimizing selection bias. Preparation of the test and the placebo substance and the analysis of final blood sugar levels were done strictly in a double-blind manner.

In conclusion,* Coccinia grandis* has a blood sugar lowering effect. Further studies are needed to extract the active component of the herb, explore the mechanism of action, and determine the dosage of the drug and the side effects. 

However following points can be mentioned as the limitations of our study. That is, we have not measured the BMI, exact concentration of the active component of *Coccinia grandis* in the study population. We also have not explained the reason for the significant difference of the sex between two groups.

## Figures and Tables

**Table 1:** Means and standard deviations of blood sugar levels for experimental and control groups before and after intervention.

Blood sugar (mg/dL)					
Groups	Mean	SD	Mean difference	95% Confidence interval	*P* value
Fasting					
Experimental (*n* = 61)	88.9	9.7			
Control (*n* = 61)	91.5	17.6	2.57	−2.49 to 7.64	0.31

One hour postprandially					
Experimental (*n* = 60)	101.3	34.7			
Control (*n* = 60)	120.8	49.0	20.2	4.81 to 35.5	0.01

Two hours postprandially					
Experimental (*n* = 59)	90.3	19.7			
Control (*n* = 60)	101.8	35.5	11.46	1.03 to 21.9	0.03
